# Neuromorphic Technologies for Neuroengineering: From Adaptive Stimulation to SNN-Based Inference and Deployable Biointerfaces

**DOI:** 10.3390/s26103049

**Published:** 2026-05-12

**Authors:** Zhengdi Sun, Anle Mu, Fuxiang Hao, Hang Wang

**Affiliations:** School of Mechanical Engineering, Xi’an University of Technology, Xi’an 710048, China

**Keywords:** neuromorphic neuroengineering, event-driven sensing, spiking neural networks, neurostimulation, sensory biointerfaces, biosignal processing, wearable and implantable systems

## Abstract

Neuromorphic technologies are attracting increasing interest in neuroengineering, as they provide an event-driven, spike-based computational framework that is well suited to temporally structured, sparse, and resource-constrained biological systems. Compared with conventional computing pipelines, neuromorphic approaches enable tighter integration of sensing, encoding, inference, feedback, and actuation under low-power and low-latency conditions. These features make them particularly relevant for wearable, implantable, and other edge-native neuroengineering applications. This review examines neuromorphic neuroengineering from four closely related perspectives: neuromorphic neurostimulation and adaptive actuation; tactile and sensory biointerfaces; spiking neural network (SNN)-based biosignal processing and state decoding; and wearable or implantable neuromorphic platforms. Across these domains, we highlight how neuromorphic systems may facilitate edge-native, closed-loop architectures that operate closer to the body and respond selectively to meaningful state changes. Neurorehabilitation is further discussed as an important translational context, as it involves long-term use, multimodal sensing, adaptive intervention, and substantial real-world deployment constraints. At present, however, the evidence base remains fragmented and is still largely dominated by device demonstrations and proof-of-concept studies rather than robust translational validation. Overall, neuromorphic approaches offer a promising systems-level pathway toward neuroengineering platforms that are not only computationally efficient but also adaptive, deployable, and responsive in real-world settings.

## 1. Introduction

Neuroengineering systems are increasingly expected to move beyond offline signal analysis and operate as closed-loop platforms that sense, interpret, and respond during use. This shift is evident across neural interfacing, bioelectronic medicine, neuroprosthetics, assistive systems, and neurorehabilitation, where practical devices must monitor physiological states, identify temporally meaningful changes in real time, and deliver feedback or intervention with minimal delay [1,2]. The engineering constraints are often severe. In wearable and implantable settings, sensing, communication, and computation cannot always be continuously offloaded to external systems, and more of these functions must instead be performed close to the body under limits on power consumption, latency, hardware footprint, and long-term usability.

Contemporary neuroengineering pipelines often begin with multichannel, high-sampling-rate biosignals, including EEG, EMG, peripheral neural recordings, pressure distributions, and other multimodal sensor streams, which are then processed using advanced machine learning and deep learning techniques [3,4]. These frameworks have substantially improved decoding performance and system functionality, but they are still predominantly implemented on conventional von Neumann architectures. Continuous sampling, repeated memory transfer, centralized numerical processing, and dense computation can increase energy consumption, introduce additional latency, and create frequent reliance on external computing resources [1,5,6,7]. This creates a practical mismatch for near-body or embedded neuroengineering systems, whose operation must be low-power, low-latency, low-maintenance, and sustainable over long durations.

At the same time, many neurophysiological and behavioral processes of interest are not uniformly informative over time. In practical scenarios, clinically or functionally meaningful information is often concentrated in salient transitions, such as motor intention onset, tactile contact events, gait-phase switching, fatigue emergence, pathological bursts, or shifts in sensorimotor engagement [8,9]. These signals are also nonstationary: their characteristics may change across contexts, sessions, and users. As a result, fixed-rate, frame-based computation is not always the most natural fit for systems that must remain robust during prolonged real-world operation. These characteristics motivate sensing and computing frameworks that respond selectively to informative events, preserve temporal structure more directly, and adapt efficiently under constrained hardware conditions [9,10].

Neuromorphic technology offers one such framework by aligning sensing and computation more closely with the principles of biological information processing. Rather than treating sensing, memory, and computation as strictly separated stages, neuromorphic systems represent and process information through spikes or spike-like events, distributed local states, and event-driven dynamics [11]. In practice, this paradigm includes synapse-like devices for local state retention and plasticity, neuron-like units for event-driven integration and nonlinear activation, and network-level models, often implemented as spiking neural networks (SNNs). When deployed on neuromorphic hardware, these elements support parallel, asynchronous, and in-/near-memory computation, reducing redundant data movement and allowing temporal information to be processed natively rather than reconstructed from densely sampled streams [12,13,14,15]. These properties make neuromorphic approaches particularly relevant to neuroengineering scenarios that require rapid responses to salient state changes, strict energy and latency constraints, and long-term autonomous operation [12,16].

**Figure 1:**
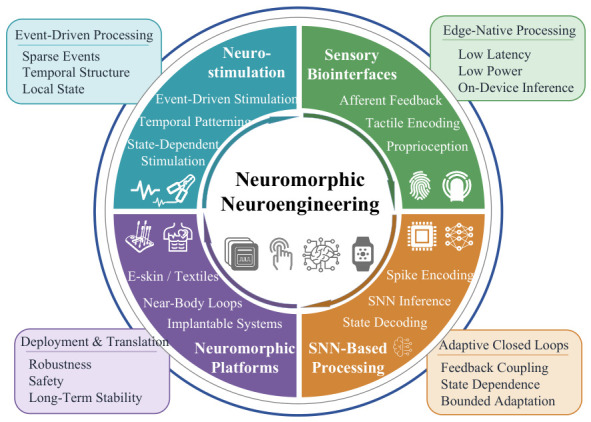
System-level framework of neuromorphic neuroengineering. The field is organized around four dimensions: neuromorphic neurostimulation and adaptive actuation, tactile and sensory biointerfaces, SNN-based biosignal processing and state decoding, and wearable or implantable neuromorphic platforms.

Importantly, the significance of neuromorphic technology in neuroengineering extends beyond energy efficiency alone. Its broader value lies in offering a systems-level logic for linking sensing, encoding, inference, feedback, and actuation under resource-constrained conditions. This becomes especially relevant when biological signals are weak, noisy, nonstationary, and only intermittently informative. In such settings, event-driven sensing, spike-based temporal representation, local state retention, and near-sensor or near-body processing can help closed-loop systems respond selectively to meaningful physiological or behavioral changes as they occur [1,2]. This relevance is reflected in several key directions in contemporary neuroengineering, including adaptive neurostimulation, bidirectional sensory biointerfaces, real-time biosignal decoding, and wearable or implantable closed-loop systems [17,18,19]. As illustrated in Figure 1, the landscape of neuromorphic neuroengineering can be organized into four closely related and mutually reinforcing dimensions: neuromorphic neurostimulation and adaptive actuation; tactile and sensory biointerfaces; SNN-based biosignal processing and state decoding; and wearable or implantable neuromorphic platforms. Together, these dimensions show that neuromorphic engineering is not merely a hardware substitute for conventional computation but a framework for reorganizing how biological signals are sensed, encoded, processed, and coupled back to the body in resource-constrained neuroengineering systems.

Neurorehabilitation, particularly in disorders involving long-term motor or sensorimotor dysfunction, represents one important translational scenario in which these properties may be especially beneficial. Nevertheless, the relevance of neuromorphic technology is not limited to rehabilitation and should be understood within the broader development of future neuroengineering systems. Against this background, this review summarizes recent advances in neuromorphic neuroengineering with emphasis on the four dimensions outlined above. By bringing these directions together within a unified framework, the review highlights how neuromorphic technologies may reshape the integration of sensing, computation, feedback, and deployment in emerging closed-loop systems. It further discusses the opportunities of these approaches for low-power, temporally adaptive, and biologically compatible biointerfaces, while identifying current evidence gaps and key challenges for future translation.

## 2. Foundations of Neuromorphic Neuroengineering

Neuromorphic neuroengineering is grounded in the observation that many neural, physiological, and biomechanical signals are inherently temporally structured, whereas the information most relevant for sensing, inference, and control is often concentrated in sparse, state-dependent changes. In practical neuroengineering scenarios, meaningful updates may correspond to motor intention onset, contact transitions, gait-phase switching, abrupt changes in muscle activation, or shifts in physiological state. This mismatch between densely sampled data streams and sparsely informative events motivates the development of computational frameworks that preserve temporal structure while reducing redundant processing [20,21,22]. From this perspective, neuromorphic approaches are attractive not only because of their potential for energy efficiency but also because they provide a computational substrate that is better aligned with real-time, resource-constrained, and near-body neuroengineering systems.

**Figure 2:**
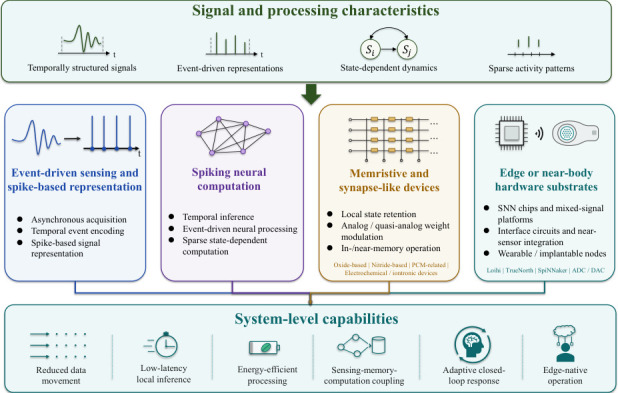
Core framework of neuromorphic neuroengineering. The figure illustrates how signal and processing characteristics give rise to the four foundational elements discussed in Section 2: event-driven sensing and spike-based representation; spiking neural computation; memristive and synapse-like devices; and edge or near-body hardware substrates. Collectively, these elements enable key system-level capabilities, including reduced data movement, low-latency local inference, energy-efficient processing, adaptive closed-loop response, and edge-native operation.

Neuromorphic technology addresses this mismatch by organizing sensing and computation around temporally structured signals, event-driven representations, state-dependent dynamics, and sparse activity patterns. As illustrated in Figure 2, these signal and processing characteristics motivate four closely related foundational elements of neuromorphic neuroengineering: event-driven sensing and spike-based representation; spiking neural computation; memristive and synapse-like devices; and edge or near-body hardware substrates. Together, these elements support system-level capabilities such as reduced data movement, low-latency local inference, energy-efficient processing, sensing–memory–computation coupling, adaptive closed-loop response, and edge-native operation [13,14,15,16].

Their relevance to neuroengineering is therefore functional rather than merely conceptual. Event-driven sensing addresses the need to detect sparse yet meaningful changes in neural, physiological, or biomechanical signals; SNN-based computation enables temporally aware and low-latency inference; memristive and synapse-like devices provide pathways for local state retention and sensing–memory–computation coupling; and edge or near-body hardware substrates enhance compatibility with wearable, implantable, and closed-loop deployment.

These foundational components form the basis for the following subsections, which examine sensing-to-spike representation, temporal computation with SNNs, device-level synaptic substrates, and hardware pathways for edge and near-body neuroengineering.

### 2.1. Event-Driven Sensing and Spike-Based Representation

Event-driven sensing is based on the premise that many biosignals and interaction signals are not uniformly informative over time. Signals such as EEG, EMG, peripheral neural activity, pressure distributions, tactile streams, and movement-related sensor outputs may evolve continuously; however, system-level decisions often depend primarily on salient transitions rather than on every sampled point. In neuroengineering tasks, these transitions may include intention onset, contact formation or release, fatigue-related shifts, or abnormal physiological events [23,24,25].

Spike-based representation provides an efficient means of encoding such changes. Instead of preserving every sample explicitly, the signal is converted into temporally localized events, whereby information is conveyed through spike timing, rate, or pattern. Recent reviews on artificial sensory neurons and neuromorphic sensory systems emphasize that sensing and spike conversion can increasingly be performed within the same front-end substrate. This integration reduces power consumption and data-transfer overhead while preserving temporally meaningful structure [12,26]. Related demonstrations, such as neuromorphically engineered tactile scanners, further illustrate how mechanical stimuli can be transformed into neuron-like tactile signals for closed-loop tactile acquisition and rendering [27].

However, the effectiveness of event-driven sensing depends critically on robust event definition. In real-world deployments, factors such as threshold selection, signal drift, movement artifacts, sensor displacement, and user-specific variability may lead to unstable or false event generation [28]. Accordingly, event-driven front-end systems should be evaluated not only in terms of detection performance but also with respect to robustness under varying operating conditions and their downstream impact on inference or control. Recent biomedical SNN studies have further highlighted the importance of adaptive threshold-based temporal encoding. For example, multimodal biosignal analysis for assessing chronic lower back pain demonstrates that robust encoding design is as crucial as the downstream model itself [29].

### 2.2. Spiking Neural Networks and Temporal Computation

Spiking neural networks (SNNs) constitute the primary computational framework of many neuromorphic systems, as they process information in a form that is inherently compatible with events and temporal dynamics. Unlike conventional neural networks, which typically operate on static vectors or frame-based inputs, SNNs evolve through neuron-like state dynamics, including membrane integration, thresholding, and refractory behavior [1,8,20]. As a result, temporal information is preserved within the computational process itself, rather than being introduced only after explicit segmentation.

A central feature of SNNs is temporal coding, whereby information may be represented through firing rate, latency, inter-spike intervals, synchrony, or distributed spatiotemporal patterns. This makes SNNs particularly well suited to tasks in which timing carries functional significance, such as neural decoding, physiological monitoring, and state-triggered control. Recent biomedical reviews have highlighted their effectiveness in processing EEG, ECG, and EMG signals, where temporal structure is often as important as signal amplitude [21]. Representative studies further demonstrate that SNNs can approach the performance of conventional deep learning models in tasks such as seizure detection and arrhythmia classification while offering more efficient temporal inference [22,23]. In addition, their event-triggered update mechanism reduces unnecessary computation when inputs are sparse, making them particularly attractive for low-latency and energy-constrained inference, especially on neuromorphic hardware [24,25].

Nevertheless, the significance of SNNs should not be interpreted solely in terms of energy efficiency. More fundamentally, they provide a computational bridge between event-based sensing and adaptive system response by treating time as a first-class variable in inference. In neuroengineering, their practical value therefore depends not only on recognition accuracy but also on latency, robustness, adaptability, and performance under realistic, noisy, and nonstationary conditions [1,20]. From this perspective, SNNs are best understood not merely as efficient classifiers but as temporal inference models that facilitate the transition from sparse sensing to closed-loop response.

### 2.3. Memristive and Synapse-like Devices for Neuromorphic Neuroengineering

At the device level, neuromorphic neuroengineering increasingly draws on memristive and other synapse-like elements that blur the conventional separation among sensing, memory, and computation. Their importance lies less in material novelty per se than in their ability to support local state retention, analog or quasi-analog weight modulation, and in-/near-memory processing within compact hardware substrates [26,27]. This systems-level role is particularly relevant in neuroengineering, where communication overhead directly affects latency, power consumption, form factor, and long-term deployability in wearable, near-body, and implantable settings.

Importantly, memristive and synapse-like devices do not constitute a homogeneous class. Representative families include oxide-based memristors, nitride-based artificial synaptic devices, phase-change-memory-related synaptic devices, and electrochemical or iontronic transistor-like synaptic elements [30,31,32,33]. As summarized in Table 1, these device families differ in their dominant switching or modulation mechanisms, endurance and retention characteristics, conductance update behavior, and scaling or integration trajectories [34,35]. From a neuroengineering perspective, such differences are not merely materials-level distinctions; rather, they determine whether a given device is better suited for dense synaptic storage, in-memory computation, sensing–memory coupling, or biologically interfaced adaptive signal processing.

Recent studies further indicate that memristive and synapse-like hardware can support several neuromorphic computing paradigms beyond static memory usage. For example, wafer-scale passive crossbar circuits highlight the potential of dense memristive integration for large-scale neuromorphic architectures [28], while memristive nanofluidic diodes illustrate alternative device-level implementations for reservoir-like computation [29]. Related studies in sensory systems further suggest that some devices can function not only as local memory units but also as adaptive sensing elements that integrate transduction and preprocessing within the same substrate [36,37]. These examples indicate that memristive devices may serve not only as programmable weights but also as local preprocessing elements that shorten the pathway from physical signal acquisition to near-memory computation. This capability is particularly relevant for neuroengineering, where wearable or implantable systems must reduce data movement while preserving temporally meaningful state information.

The principal system-level advantage of such devices lies in the co-location of memory and computation, thereby reducing repeated data transfer among sensors, memory, and processors. This is especially valuable in neuroengineering systems operating under stringent constraints on energy, latency, and maintenance burden [26,27,34]. At the same time, practical translation remains constrained by device non-idealities, including variability, drift, finite endurance, imperfect analog weight updates, and calibration overhead [12,34,35]. These limitations become particularly critical when memristive or synapse-like elements participate directly in sensing, inference, or adaptive control, as instability at the device level may propagate to system-level decisions rather than remaining a purely materials-level concern. Accordingly, in neuromorphic neuroengineering, the key question is not only whether synaptic behavior can be emulated but also whether such devices can achieve sufficient stability, reproducibility, and operational lifetime to support reliable closed-loop deployment.

### 2.4. Hardware Substrates and Interface Pathways for Edge and Near-Body Neuroengineering

The system-level relevance of neuromorphic principles becomes most evident when they are implemented on hardware platforms that place sensing, encoding, memory, inference, and, in some cases, preliminary control close to the locations where signals are generated or interventions are required. In neuroengineering, this hardware perspective is particularly important because wearable, patch-based, textile-integrated, and implantable systems operate under stringent constraints on latency, power consumption, communication bandwidth, form factor, and long-term autonomy. From this viewpoint, neuromorphic neuroengineering depends not only on algorithms or devices in isolation but also on the specific hardware substrates that support event-driven computation under near-body deployment conditions.

Representative neuromorphic platforms that currently dominate the field include Loihi, Loihi 2, TrueNorth, and SpiNNaker, as summarized in Table 2. These platforms differ in architecture, scale, programmability, and reported power or energy characteristics, but all aim to reduce the cost of temporal processing by combining event-driven communication with hardware structures tailored to spiking computation [38,39,40,41,42,43]. In relation to the application scenarios discussed elsewhere in this review, platforms in the Loihi family are particularly relevant for adaptive and low-latency edge inference, as they support programmable spiking dynamics and on-chip learning or signal-processing-oriented extensions [38,42]. TrueNorth exemplifies ultra-low-power, large-scale neurosynaptic integration and is therefore representative of hardware directions targeting highly energy-constrained neuromorphic sensing and classification [40,41]. SpiNNaker, in contrast, emphasizes large-scale, real-time spiking neural network simulation through massively parallel digital hardware and packet-switched communication, making it especially relevant for neuroengineering applications that require larger closed-loop network models or flexible real-time neural simulation [41,43].

At the same time, neuromorphic hardware should not be understood solely in terms of named digital platforms. Related chip-level developments in in-memory computing, analog phase-change-memory-based systems, and mixed-signal neuromorphic architectures further expand the hardware landscape [32,34,44]. These approaches address a different but equally critical challenge: the cost of repeated data transfer between memory and compute units. In-memory and mixed-signal strategies aim to move multiply–accumulate operations, state storage, or aspects of analog signal transformation closer to the physical memory substrate, thereby reducing data movement overhead and improving hardware efficiency. From a neuroengineering perspective, such approaches are particularly attractive for near-body systems that must continuously process temporally structured data under limited power and area budgets.

A critical hardware-level issue, however, is that the efficiency of a neuromorphic processor cannot be evaluated in isolation from its interface circuitry. In near-sensor systems, analog front-end acquisition is typically followed by signal conditioning, analog-to-digital conversion, event extraction, neuromorphic or digital processing, and, in some cases, digital-to-analog conversion for stimulation or output control. The converters and peripheral circuits within this pathway can contribute substantially to system-level power consumption, area, and latency [34,45,46]. Recent work in computing-in-memory has explicitly highlighted that the widespread use of ADCs and DACs in mixed-signal accelerators can significantly erode the gains of memory-centric computing, thereby motivating architectures that reduce or localize such conversions [46]. Related demonstrations of reconfigurable analog hardware further suggest that ADC/DAC functions may themselves become part of more adaptive circuit pathways rather than remaining fixed external interfaces [47]. For neuromorphic neuroengineering, this implies that near-sensor intelligence should be evaluated at the level of the complete processing pathway—from the analog front end through event generation and computation to output interfacing—rather than solely at the processor core.

These hardware concepts are also beginning to appear in near-body neuromorphic systems with increasingly application-specific form factors. For example, neuromorphic electronic skin has been used to integrate event-driven sensing with local perception of injury-related stimuli [48], while neuromorphic multifunctional sensing fibers demonstrate that textile- or fiber-based substrates can support wearable human–machine interaction with embedded intelligent processing [49]. Implantable systems extend this paradigm to scenarios requiring highly localized interfacing or long-term physiological monitoring, but they also impose stricter requirements on mechanical compliance, encapsulation, biocompatibility, and long-term stability [50]. In this context, the practical value of neuromorphic hardware in neuroengineering depends not only on nominal computational efficiency but also on how effectively device arrays, processing cores, communication fabrics, memory units, and interface circuits are integrated into robust near-body processing pathways.

Overall, current hardware developments indicate that neuromorphic neuroengineering is shaped not only by spike-based computation but also by the manner in which hardware substrates and interface pathways bring temporal processing closer to the body. Accordingly, the practical significance of these platforms depends not only on processor-level efficiency but also on how effectively they reduce data movement, support temporally structured processing, and remain compatible with the power, size, and stability requirements of real-world neuroengineering systems.

## 3. Neuromorphic Neurostimulation and Adaptive Actuation

Neuromorphic neurostimulation reframes stimulation from a fixed waveform prescription into an adaptive intervention process, in which temporal structure, state dependence, and closed-loop updating become central design variables. In conventional neurostimulation practice, electrical stimulation is often categorized according to target as functional electrical stimulation (FES) or neural stimulation, including both central and peripheral approaches [51,52,53,54,55]. FES primarily aims to elicit muscle contraction for task execution, whereas neural stimulation more directly modulates excitability and circuit dynamics.

Across both categories, however, many established protocols continue to rely on regular, stationary pulse trains that only coarsely approximate the stochastic, sparse, and context-dependent firing patterns observed in biological motor pathways. This limitation has driven increasing interest in biomimetic or neuromorphic stimulation strategies that more closely align stimulation with physiological coding principles. The objective is to improve recruitment selectivity, temporal responsiveness, and engagement of plasticity-related mechanisms while maintaining compatibility with safety-constrained clinical use [56,57].

### 3.1. Why Temporal Structure Matters in Neurostimulation

A central premise of neuromorphic neurostimulation is that the effects of stimulation depend not only on amplitude, frequency, and target location but also on temporal patterning. Biological motor pathways do not typically operate through perfectly regular pulse trains; instead, their activity is characterized by irregular firing, burst-like behavior, rhythm-dependent modulation, and context-sensitive temporal transitions. Accordingly, stimulation strategies based on Poisson-like statistics, burst structures, irregular interpulse intervals, and rhythm-aligned trains have been explored as approaches to better approximate natural firing dynamics and to provide richer temporal degrees of freedom for modulating recruitment and plasticity-related processes [56,57].

**Figure 3:**
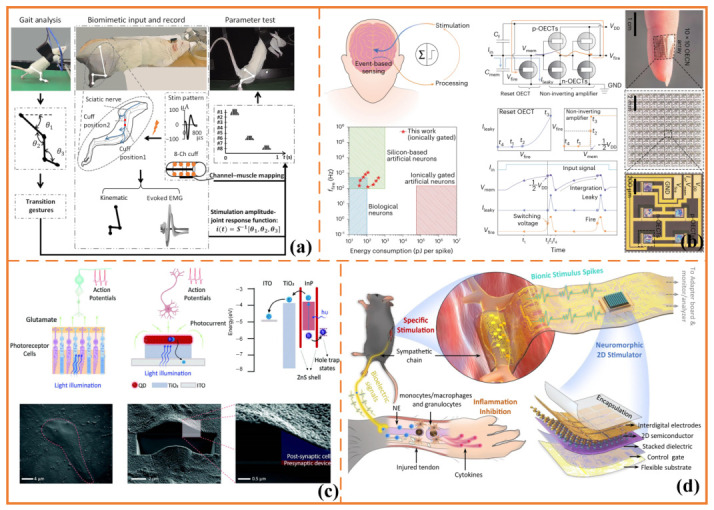
Representative examples of neuromorphic neurostimulation and adaptive actuation. (**a**) Biomimetic peripheral nerve stimulation for hindlimb modulation during stepping, adapted from Xi et al. [61] under a Creative Commons Attribution 4.0 (CC BY 4.0) license; (**b**) organic event-based sensing for low-energy closed-loop neurostimulation, adapted from Yang et al. [62] under a CC BY 4.0 license; (**c**) retina-inspired optoelectronic synapse for neuromorphic photostimulation, adapted from Balamur et al. [63] under a CC BY 4.0 license; (**d**) direct nerve neuromorphic electro-stimulation using an atomically thin semiconductor floating-gate-memory stimulator, adapted from Bao et al. [64] under a CC BY 4.0 license. Visual annotations, including arrows, colors, and lines, are retained from the corresponding source panels and indicate signal pathways, stimulation routes, device structures, or schematic relationships in the respective studies.

This principle has been investigated in both central and peripheral contexts. In central stimulation, invasive modalities such as deep brain stimulation (DBS) provide access to distributed motor-related circuits. Exploratory post-stroke applications, including cerebellar dentate nucleus stimulation, suggest potential relevance for motor recovery; however, the available evidence remains limited, and protocol standardization is still lacking [58,59,60]. In peripheral systems, temporally structured stimulation aligned with locomotor rhythms has also been examined in animal models. As shown in Figure 3a, biomimetic peripheral nerve stimulation has been used to characterize relationships between sciatic nerve branches and hindlimb muscle recruitment, as well as to modulate stepping-related joint movements in rats. These findings support the feasibility of temporally shaped recruitment under controlled stimulation conditions [61].

At present, this body of work is best interpreted as providing mechanistic insight and feasibility evidence rather than definitive proof of clinical superiority.

### 3.2. Event-Driven and State-Dependent Stimulation

A second defining feature of neuromorphic stimulation is the transition from open-loop delivery to event-driven and state-dependent actuation. In this framework, stimulation is updated when salient physiological or behavioral events occur—such as gait-phase transitions, intention-related state changes, or shifts in peripheral or cortical activity—rather than being delivered continuously according to a fixed schedule. The conceptual significance of this shift lies not only in reduced computational cost but also in tighter alignment between intervention timing and the evolving biological state of the system. As shown in Figure 3b, recent work has demonstrated that high-frequency, low-energy organic event-based sensors can support rapid neural signal detection and closed-loop neurostimulation. In particular, organic electrochemical neuron-based sensors have been reported to operate at millisecond-scale response speeds, generate spike-like pulses across the physiological neural bandwidth, and enable real-time stimulation to suppress pathological oscillatory activity in vivo. These findings illustrate how event-driven hardware may strengthen the coupling between sensing and stimulation in implantable neurotherapeutic systems [62].

Beyond sensor-triggered stimulation, compact neuromorphic interfaces have also begun to integrate sensing, memory, and stimulation within a single device architecture. As shown in Figure 3c, a retina-inspired optoelectronic synapse based on quantum dots exemplifies this trend by combining photodetection, synaptic memory, and neuronal photostimulation within a unified interface [63]. This design is particularly significant because it suggests that stimulation interfaces themselves may embody event-driven sensing and short-term plasticity-like dynamics, rather than functioning solely as passive output stages.

Representative work further includes closed-loop FES or pathway stimulation informed by peripheral neural recordings, supporting the feasibility of linking neural activity, stimulation, and motor output within adaptive loops [65]. In parallel, neuromorphic control strategies—particularly those involving SNN-based temporal processing—have been explored to exploit sparse event-driven updates within the stimulation loop and to improve recruitment selectivity or control precision under hardware constraints [66]. Studies in stroke-related animal models further suggest that stimulation parameters can be adjusted according to residual cortical activity, while related peripheral investigations have examined temporally structured locomotor stimulation in state-dependent settings [67].

Taken together, these efforts shift the role of stimulation from that of a static waveform generator toward that of an adaptive actuator embedded within a sparse, temporally responsive control loop.

### 3.3. System Integration Requirements

The practical value of neuromorphic neurostimulation depends not only on stimulation pattern design or adaptation logic but also on system-level integration. In real-world neuroengineering settings, adaptive stimulation requires reliable coupling between sensing and actuation, sufficient timing fidelity for state-triggered updates, controllable multi-electrode routing, and robust long-duration operation. Spatially distributed recruitment may depend on precise relative timing across channels, particularly in multi-electrode systems designed to compose spatiotemporal activation patterns rather than deliver isolated pulses [68]. As shown in Figure 3d, recent work on neuromorphic electrostimulation has further illustrated how device-level memory behavior can be coupled with stimulation output. Bao et al. developed an atomically thin MoS_2_ floating-gate memory interdigital circuit capable of generating programmable bionic stimulation spikes for direct sympathetic-chain stimulation. This approach suggests a pathway toward stimulation interfaces in which local state retention, programmable stimulation dynamics, and electrical actuation are integrated within a single device-level platform [64].

In addition, power delivery, packaging, and long-term operational stability are critical determinants of whether adaptive stimulation can be implemented beyond short-duration laboratory demonstrations. These considerations include advances in neuromorphic stimulation interfaces, power management strategies, and biomimetic microenvironments or scaffolds that interact with tissue and regenerative processes [64,69]. For example, electroconductive MXene-based micro-meshes embedded within a biomimetic hyaluronic-acid scaffold illustrate how structural biointerfaces may influence stimulation delivery in neural repair contexts [69]. From a translational perspective, these elements function as deployment enablers: they do not replace control algorithms, but they strongly influence whether closed-loop stimulation can be delivered safely, consistently, and with an acceptable maintenance burden.

System validation should therefore extend beyond waveform parameters alone. For neuromorphic stimulation systems, relevant performance indicators include sensing–stimulation latency, state-transition reliability, timing precision, recruitment controllability, safety-related events, and long-term tolerance. Such metrics are particularly important when stimulation is updated online in response to sparse physiological events, as instability in sensing, timing, or control may directly affect both efficacy and safety [61,70]. To facilitate cross-study comparison, representative work in neuromorphic neurostimulation and adaptive actuation is summarized in Table 3, with emphasis on target scenario, neuromorphic features, loop level, and stage of evidence.

### 3.4. Relevance to Motor Restoration and Neurorehabilitation

From the perspective of motor restoration, the primary contribution of neuromorphic neurostimulation lies not merely in the introduction of alternative stimulation waveforms, but in the redefinition of stimulation as a temporally structured and state-linked actuation process. Within this framework, stimulation is no longer treated as an isolated output command; instead, it becomes embedded within a loop in which sensing, event detection, timing control, and intervention are more tightly integrated. This shift is particularly relevant for motor systems, where the functional effects of stimulation depend strongly on when it is delivered, how it is patterned, and whether it remains aligned with ongoing physiological or behavioral state changes.

For neurorehabilitation, this systems-level perspective is more consequential than any single device demonstration. The most significant implication of the studies reviewed here is that stimulation can increasingly be embedded within adaptive sensing–stimulation loops, including event-triggered neuromodulation, bidirectional peripheral interfaces, and self-powered or biointegrated stimulation platforms [62,65,71]. In other words, the field is progressively transitioning from fixed stimulation delivery toward architectures in which temporally relevant information determines when and how stimulation is applied. This distinction represents a central insight of the current literature, as it clearly differentiates neuromorphic stimulation from conventional open-loop pulse delivery.

At the same time, the strongest evidence to date primarily supports the feasibility of coupling, triggering, and system integration, rather than demonstrating definitive rehabilitative superiority. Many reported systems remain at the stage of device validation, preclinical animal studies, or early closed-loop demonstrations, while evidence for long-term stability, cross-condition robustness, and deployment-ready operation remains limited [60,61,65,66,67]. For this reason, the next phase of development in neuromorphic neurostimulation should focus not only on proposing novel stimulation patterns but also on determining the conditions under which adaptive sensing–stimulation loops remain reliable, safe, and functionally effective over prolonged use. Such progress will be essential if neuromorphic stimulation is to evolve from an innovative control paradigm into a practical component of restorative and rehabilitative neuroengineering systems.

## 4. Neuromorphic Tactile and Sensory Biointerfaces

Neuromorphic tactile and sensory biointerfaces are motivated by the need to restore or strengthen the coupling between mechanical interaction and afferent signaling in human–machine systems. Tactile and proprioceptive feedback are essential for object manipulation, posture regulation, and sensorimotor coordination; however, conventional biointerface designs often emphasize intention decoding or motor actuation while under-specifying the sensory return pathway. In such cases, interaction mechanics may be measured, but the resulting feedback is frequently reduced to regular pulse trains or low-dimensional control variables that do not capture the sparse, asynchronous, and temporally structured nature of biological afferent signaling [72,73,74].

Neuromorphic sensory approaches address this limitation by treating sensory interfacing as a complete processing chain, in which mechanical stimuli are transformed into spike-compatible representations and subsequently delivered through temporally structured feedback or stimulation. From this perspective, these systems are best understood not as isolated tactile devices, but as integrated biointerface architectures that support bidirectional sensorimotor communication [75,76,77].

### 4.1. Biomimetic Sensory Front Ends

**Figure 4:**
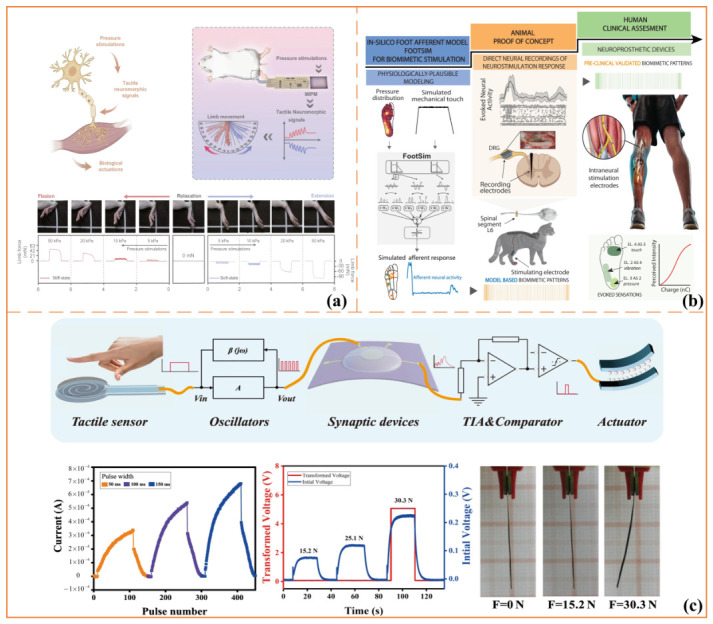
Representative examples of neuromorphic tactile and sensory biointerfaces. (**a**) Mechano-gated iontronic piezomemristor for temporal-tactile neuromorphic plasticity, adapted from [81] under a Creative Commons Attribution 4.0 (CC BY 4.0) license; (**b**) biomimetic encoding-to-stimulation pathway for naturalistic touch via peripheral nerve stimulation, adapted from [82] under a CC BY 4.0 license; (**c**) artificial neuromorphic somatosensory system with spatio-temporal tactile perception and instant feedback functions, adapted from [83] under a CC BY 4.0 license. Arrows and colors in the adapted panels are retained from the corresponding source figures and indicate stimulation directions, signal pathways, sensing processes, or schematic relationships in the respective studies.

A central direction in neuromorphic sensory interfacing is the development of biomimetic front ends that emulate the response characteristics of biological mechanoreceptors. Rather than relying solely on simple amplitude–frequency coding, recent systems increasingly seek to reproduce adaptation dynamics, time constants, and differential sensitivity to static and dynamic touch [78,79]. For example, artificial afferent systems that integrate pressure-activated mechanoreceptors with synapse-like transistors have been used to distinguish sustained from transient contact and to support closed-loop slip recognition and prevention in robotic manipulation [80]. As shown in Figure 4a, related work has further demonstrated that mechano-gated iontronic piezomemristive structures can convert complex pressure inputs into positive and negative spike trains, thereby generating excitatory and inhibitory neuromorphic responses with temporal tactile plasticity [81].

Together, these studies demonstrate how tactile stimuli can be locally transformed into neuromorphic signals prior to downstream processing or feedback, thereby enhancing compatibility with event-driven information flow. Within neuroengineering systems, such front ends are particularly significant because they constitute the interface between interaction mechanics and neural-like information processing. Pressure arrays, tactile sensors, electronic skin (e-skin) platforms, and other conformable sensing structures can therefore be understood not merely as measurement devices, but as the initial layer of a sensory pathway that extracts temporally meaningful events from physical contact.

This capability is especially relevant in applications requiring low power consumption and real-time interaction, as biomimetic front ends enable mechanical signals to be translated into sparse event streams prior to subsequent encoding, inference, or feedback generation.

### 4.2. Encoding-to-Stimulation Pathways

A second major direction concerns the design of encoding-to-stimulation pathways that preserve temporal structure from the sensing stage to the feedback stage. In this context, the objective extends beyond simply detecting contact or force magnitude; rather, it involves converting measured interaction mechanics into spike-like representations and using these representations to generate stimulation patterns that more closely approximate natural afferent firing [76,77]. Within this framework, sensory biointerfaces function as pipelines that map external mechanical inputs to neural-like feedback codes, rather than as controllers that independently adjust stimulation amplitude or frequency.

Representative work includes both computational and hardware-assisted approaches for deriving stimulation patterns from predicted afferent activity. For example, FootSim provides a structured workflow in which plantar mechanical input is transformed into simulated spiking responses of the four major classes of foot-sole mechanoreceptive afferents, thereby offering an in-silico basis for the design of biomimetic stimulation sequences [84]. As shown in Figure 4b, studies building on this modeling paradigm further demonstrate that biomimetic stimulation policies derived from physiologically plausible afferent codes can elicit neural responses that more closely resemble those evoked by natural touch than conventional regular-pulse stimulation [82].

The significance of this direction is therefore both methodological and technological: it establishes a reproducible bridge between measured interaction mechanics and physiologically informed feedback codes, thereby supporting more coherent sensory pathway design in closed-loop biointerfaces [85,86].

### 4.3. Tactile and Proprioceptive Feedback in Bidirectional Interfaces

Beyond front-end sensing and encoding, neuromorphic sensory systems are increasingly relevant to bidirectional interfaces in which tactile and proprioceptive feedback reinforce motor interaction. In such systems, the sensory pathway is not merely an auxiliary component, but a functionally integral part of the loop linking action, contact, perception, and subsequent adjustment. This distinction is particularly important because tactile and proprioceptive information are not equivalent: cutaneous inputs can often be approximated through localized pressure- or vibration-based encoding, whereas proprioception depends on multiple receptor classes, musculoskeletal state, and more distributed central integration [87,88,89]. As a result, generalized, one-code-fits-all sensory stimulation is unlikely to provide sufficiently rich or stable feedback across tasks and users.

Selective stimulation of peripheral sensory pathways may, in principle, support the restoration or substitution of tactile and proprioceptive inputs [90,91]; however, practical implementation remains constrained by electrode count, safe stimulation limits, mapping stability, and the need to preserve perceptual interpretability over time. Representative studies further indicate that proprioceptive restoration requires more than the delivery of patterned stimulation alone. For example, ProprioStim provides a framework for deriving neurostimulation policies from a symbiotic electroneural and musculoskeletal model, highlighting the importance of anatomically and biomechanically grounded encoding for achieving naturalistic proprioceptive feedback [91]. Related work on low-power, stretchable neuromorphic nerves with proprioceptive feedback further demonstrates that artificial proprioceptors can be integrated with neuromorphic implants to support smoother and more coordinated motor output [89].

As shown in Figure 4c, recent work has extended neuromorphic tactile interfaces beyond perception toward integrated perception–feedback systems. An artificial neuromorphic somatosensory system has been developed by combining flexible tactile sensors, synaptic transistors, coupling circuits, and an artificial muscle, thereby enabling spatiotemporal tactile perception and immediate feedback actuation [83]. This type of system-level demonstration is particularly relevant to bidirectional interfaces, as it illustrates how tactile sensing, neuromorphic information processing, threshold-based feedback, and actuation can be coherently integrated within a closed sensory–motor pathway.

Accordingly, the value of neuromorphic sensory biointerfaces lies in their capacity to support temporally structured, task-relevant, and state-consistent feedback that operates alongside motor decoding or actuation modules, thereby strengthening the sensory dimension of closed-loop human–machine interaction [83,87,88,89,90,91,92,93]. To facilitate cross-study comparison, representative work in neuromorphic tactile and sensory biointerfaces is summarized in Table 4, with emphasis on target scenario, biomimetic features, interface roles, and stage of evidence.

### 4.4. Application Relevance: Neuroprostheses, Assistive Systems, and Rehabilitation

The primary significance of neuromorphic tactile and sensory biointerfaces lies in their renewed emphasis on the sensory component of the closed loop. Rather than treating tactile input as an auxiliary signal or a simplified control variable, the studies reviewed here increasingly conceptualize sensory interfacing as a structured pathway that links interaction mechanics to neural-like encoding and, subsequently, to feedback delivery. This shift is particularly important in neuroengineering, as functional interaction depends not only on decoding intention or generating movement but also on whether the resulting sensory consequences can be represented in a temporally meaningful and behaviorally relevant form.

From this perspective, the most robust current evidence arises from neuroprosthetic and assistive applications, where biomimetic tactile sensing and feedback have demonstrated that more structured sensory return can improve grasp control, interaction quality, and perceptual naturalness [73,82,92]. Importantly, the key contribution of this body of work is not merely the emergence of new tactile devices, but the increasing coherence and biological grounding of encoding-to-stimulation pathways. In other words, the field is progressing beyond isolated tactile sensing or ad hoc stimulation toward sensory biointerfaces that explicitly aim to preserve the relationship between contact events, afferent coding, and action-relevant feedback.

In rehabilitation contexts, however, the central question is no longer whether such sensory strategies are conceptually promising, but whether they can remain stable, interpretable, and functionally effective over prolonged training periods and under varying task conditions. Current evidence remains uneven across device demonstrations, robotic manipulation studies, computational encoding frameworks, and neuroprosthetic feasibility research [73,92]. Only a limited number of studies have systematically examined long-term perceptual consistency, cross-task robustness, or sustained contributions to sensorimotor training under realistic neuroengineering constraints, such as interface durability, stimulation stability, and evolving biomechanics. Accordingly, the next stage of development should not focus solely on improving sensory encoding in isolation, but rather on determining how neuromorphic tactile feedback can support durable, bidirectional function within fully integrated closed-loop systems.

## 5. SNN-Based Biosignal Processing and State Decoding

SNN-based processing is emerging as a critical computational layer in neuroengineering, as many neural, physiological, and interaction signals are inherently time-dependent, while the information most relevant for inference and control is often sparse, state-dependent, and embedded in temporal transitions. Compared with conventional artificial neural networks, SNNs offer intrinsic advantages in temporal coding, event-driven computation, and sparse communication. They are therefore widely regarded as a core algorithmic paradigm for neuromorphic signal-processing systems [94,95]. This is particularly relevant for biosignals such as EEG, EMG, ECG, high-density sEMG, pressure and contact streams, and event-based motion signals, where the primary challenge extends beyond pattern classification to the extraction of functionally meaningful state information under constraints of latency, computational efficiency, and long-duration operation.

By transforming continuous or discretely sampled physiological signals into spike trains and performing inference through SNN-based models, it becomes possible, in principle, to preserve task-relevant temporal structure while reducing sampling redundancy and communication overhead. This characteristic makes SNN-based processing especially suitable for edge and near-body neuroengineering systems, where biosignal decoding, state monitoring, and adaptive control must often be executed locally rather than relying on continuous external computation [96,97].

From a review perspective, the following discussion first outlines key algorithmic foundations of contemporary SNN research and then examines four closely related directions: event-driven sensing and spike-encoding front ends; SNN-based temporal inference for neural and physiological signals; edge intelligence for intention decoding and adaptive monitoring; and evaluation frameworks that extend beyond conventional accuracy-based metrics.

### 5.1. Algorithmic Foundations of SNN-Based Processing

Before considering application-specific uses in neuroengineering, it is useful to identify three core dimensions that shape contemporary SNN research: training methods, neuron models, and evaluation benchmarks. In terms of learning strategies, current SNN studies are typically organized around three broad approaches: ANN-to-SNN conversion, local plasticity rules such as spike-timing-dependent plasticity (STDP), and direct gradient-based training using surrogate gradients. ANN-to-SNN conversion remains attractive because it leverages mature ANN optimization pipelines and often achieves high accuracy with reduced latency, whereas STDP and related local rules retain biological interpretability and support hardware locality. In contrast, direct surrogate-gradient-based training has emerged as a dominant supervised learning paradigm for deep SNNs, as it enables end-to-end optimization while preserving spike-based neuronal dynamics [98,99,100].

A second foundational dimension concerns neuron models. Common SNN implementations range from simple integrate-and-fire and leaky integrate-and-fire (LIF) neurons to more expressive formulations, including the spike response model, Izhikevich neurons, and Hodgkin–Huxley-type dynamics. In practice, LIF-based models remain particularly prevalent in large-scale and hardware-oriented SNN research, as they provide a favorable balance between temporal expressiveness, computational efficiency, and implementation simplicity. Although more biophysically detailed models can capture richer neural dynamics, they also introduce higher training and simulation costs, which limit their routine use in deep SNN pipelines [100,101].

A third key dimension is benchmarking. SNN performance is commonly evaluated on both conventional static datasets and event-based neuromorphic benchmarks. Widely used datasets include CIFAR10 and CIFAR100 following spike encoding, as well as event-driven datasets such as N-MNIST, CIFAR10-DVS, DVS Gesture, and related neuromorphic vision benchmarks [101,102]. While these benchmarks are useful for comparing architectures and training strategies, they do not fully reflect the deployment constraints relevant to neuroengineering applications. Consequently, evaluation in neuroengineering-oriented SNN systems should extend beyond classification accuracy to include latency, robustness under nonstationary inputs, and, where possible, resource-aware performance under edge or near-sensor operating conditions [101,102,103].

### 5.2. Event-Driven Sensing and Spike Encoding Front Ends

At the front end of neuromorphic biosignal processing, a central objective is to convert physical or physiological signals into spike-compatible representations that can be processed in an event-driven, rather than frame-based, manner. Representative studies demonstrate that tactile, force, contact, posture, and motion-related signals can be encoded into sparse event streams and integrated with neuromorphic or neuromorphic-inspired inference modules. For example, force-sensing neuromorphic front ends have been developed in which applied mechanical pressure is directly translated into spike-like outputs through sensor–neuron integration, enabling low-power, event-driven force monitoring for robotic interaction tasks [104]. Pressure-distribution-based systems further show that posture-related states can be encoded and classified using SNNs, supporting the feasibility of sparse tactile-state inference from distributed contact information [105].

**Figure 5:**
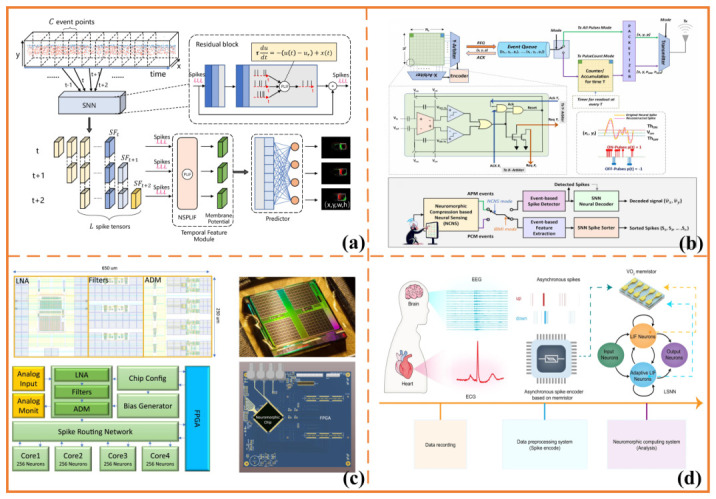
Representative examples of SNN-based biosignal processing and state decoding. (**a**) Eye tracking based on event-camera sensing and spiking neural network processing, adapted from [107] under a Creative Commons Attribution 4.0 (CC BY 4.0) license; (**b**) neuromorphic neural compression for wireless implantable brain–machine interfaces, adapted from [109] under a CC BY 4.0 license; (**c**) electronic neuromorphic detection of high-frequency oscillations in intracranial EEG, adapted from [110] under a CC BY 4.0 license; (**d**) VO_2_ memristor-based neuromorphic physiological signal processing for human–machine interfaces, adapted from [111] under a CC BY 4.0 license. Visual annotations, including arrows, colors, lines, and dots, are retained from the corresponding source panels and indicate event flows, signal pathways, neural-network modules, device structures, or schematic relationships in the respective studies.

In the visual domain, event-based cameras combined with SNN models have also demonstrated strong potential for dynamic perception tasks. Recent work includes multiscale spiking vision transformer frameworks for event-based object detection, which enhance the representation of spatiotemporal event features [106], as well as eye-tracking systems that integrate event cameras with SNN processing to achieve efficient real-time motion estimation under sparse visual input [107] (Figure 5a). Collectively, these studies support the feasibility of a “physical signal → spike encoding → event-driven inference” front-end paradigm for neuroengineering applications.

However, a critical challenge in real-world deployment is not merely whether a signal can be encoded into spikes, but whether event definitions remain stable during prolonged use and under practical conditions such as sensor displacement, perspiration or skin-impedance variation, motion artifacts, and inter-subject heterogeneity [108]. From this perspective, spike encoding should be regarded as a design problem rather than a fixed preprocessing step. Factors such as threshold drift, adaptation dynamics, and nonstationary noise can substantially alter event statistics and thereby affect downstream inference and control. Consequently, front-end evaluations should report deployment-relevant metrics, including cross-session stability of event rates, false-event burden under artifact conditions, and downstream impacts on decision-relevant outputs, rather than relying solely on static recognition accuracy [8].

### 5.3. SNN-Based Temporal Inference for Neural and Physiological Signals

Beyond the sensing layer, SNNs have been widely explored for temporal inference in neural and physiological signals, as they retain time as an explicit computational variable. A growing body of work demonstrates that EEG signals can be converted into spike trains for seizure-related event detection, achieving performance comparable to CNN- or RNN-based baselines while reducing computational cost [110] (Figure 5c). Similar approaches have been applied to ECG analysis, in which temporal features such as RR intervals and waveform morphology are encoded into spikes for multiclass arrhythmia classification, indicating a plausible pathway toward neuromorphic deployment in cardiovascular monitoring [112].

Memristor-enabled co-design frameworks further extend this direction by integrating asynchronous spike encoders with SNN-based decision modules. In particular, recent work based on VO_2_ memristors has demonstrated an end-to-end neuromorphic physiological signal-processing system, in which volatile memristive dynamics are leveraged to emulate both short- and long-term temporal responses. This approach enables the integration of signal encoding, temporal feature extraction, and classification within a unified, hardware-oriented architecture [111] (Figure 5d). The significance of this line of research lies less in isolated benchmark performance and more in its collective demonstration of the feasibility and increasing maturity of SNN-based temporal inference for noisy, time-dependent, and clinically relevant signals.

Related studies have also employed SNN-generated spasm-like EMG patterns in silico to stress-test stimulation or control systems under abnormal input conditions [113], highlighting the broader role of SNN-based processing not only in classification but also in robustness evaluation and system validation. At the same time, much of the current evidence remains centered on disease-event detection tasks, such as seizures or arrhythmias, where labels are relatively well defined. In contrast, neuroengineering applications often require more context-dependent targets, including fatigue transitions, spasticity episodes, engagement fluctuations, or safety-critical anomalies. Accordingly, this body of work should be interpreted primarily as evidence of feasibility and technological maturity in low-power temporal inference, rather than as direct validation of application-specific effectiveness. Future research will require event targets that are more closely aligned with operational neuroengineering workflows, as well as longitudinal datasets and state annotations that support temporally meaningful deployment-oriented evaluation [114,115].

### 5.4. Edge SNN-Based Inference for Intention Decoding, Monitoring, and Adaptive Control

SNN-based processing becomes most relevant to closed-loop neuroengineering when SNN models move beyond offline classification and begin to support intention decoding, state monitoring, and adaptive control under resource constraints. Existing studies demonstrate that motor-unit spike trains derived from sEMG decomposition can be processed by residual SNNs to achieve high-accuracy classification of complex hand and wrist movements in both healthy individuals and stroke survivors while substantially reducing inference energy compared with ResNet-style baselines [116]. On the neural side, SNN decoders have been applied to motor-imagery and movement-related EEG classification, and end-to-end SNN models for motor imagery have been reported to approach state-of-the-art CNN performance on public datasets while maintaining lower energy consumption [117,118,119]. Collectively, these findings indicate that SNN-based decoding is increasingly capable of supporting control-relevant inference from both peripheral and central biosignals.

At the system level, neuromorphic edge frameworks are increasingly emphasizing complete processing pipelines that span signal acquisition, preprocessing, spike encoding, compression, and on-device inference. For example, recent work on wireless implantable brain–machine interfaces has proposed a neuromorphic compression-based neural sensing architecture with address-event-representation-inspired readout, demonstrating that large-scale neural recordings can be substantially compressed while preserving spike information for downstream detection tasks [109] (Figure 5b). In parallel, other studies have demonstrated closed-loop BCI implementations on neuromorphic hardware with personalized adaptation [120,121], suggesting a broader transition from isolated SNN inference modules to deployable, edge-native control pipelines.

These developments indicate that spiking models are beginning to function as operational components within integrated neuroengineering systems, extending their role from signal classification to continuous state monitoring and adaptive control. Their relevance is particularly pronounced in edge and near-body applications, where timely and reliable decisions must be made under stringent power and latency constraints. Nonetheless, several important gaps remain, including limited longitudinal validation, insufficient assessment of cross-session drift and domain shift, and the absence of standardized labels or protocols for application-critical states [120,121]. To facilitate cross-study comparison, representative work in SNN-based biosignal processing and state decoding is summarized in Table 5, with emphasis on signal scenarios, neuromorphic features, system roles, and the current stage of evidence.

### 5.5. Evaluation Metrics Beyond Accuracy

For SNN-based processing in neuroengineering, conventional accuracy metrics are necessary but insufficient. Because many target applications involve edge deployment, state-triggered decision-making, and prolonged interaction with noisy biological systems, evaluation must also capture whether an SNN-based pipeline remains reliable, efficient, interpretable, and safe under realistic operating conditions. Relevant criteria therefore include inference latency, energy consumption, cross-session robustness, tolerance to domain shift, false-event rates, temporal precision, and the downstream consequences of failure when decoding outputs are used for stimulation, feedback, or control [96,97,114,115,120,121]. This perspective implies that future reporting standards should move beyond isolated benchmark accuracy and instead provide a joint characterization of computational efficiency, robustness, and functional impact under realistic sensing and deployment conditions.

What should be retained from the current literature is that SNN-based processing has already progressed beyond purely abstract algorithmic interest and is beginning to demonstrate its clearest value in tasks where temporal structure, sparsity, and local computation are directly coupled to deployment constraints. The strongest current evidence is concentrated in event-driven front ends, pathological-event detection, neural compression, and resource-aware edge decoding, where spike-based representations can reduce data movement and support timely inference without relying entirely on centralized processing [109,110,111,116]. In this sense, the primary contribution of the field is not that SNNs have universally outperformed conventional models but that they are increasingly functioning as operational modules within realistic sensing-to-decision pipelines.

However, a major limitation is that most available evidence still derives from short-duration demonstrations, benchmark-oriented classification tasks, or narrowly defined event targets. While many studies demonstrate that spike encoding and SNN inference are feasible, far fewer show that these approaches remain stable and decision-relevant over prolonged use, evolving physiological states, annotation uncertainty, and application-critical failure conditions [114,115,120,121]. In neuroengineering, this distinction is critical: the central question is no longer whether spiking models can classify signals efficiently but whether they can support durable monitoring, control, and adaptive decision-making as the signal, the user, and the context evolve over time. Future progress in this area will therefore depend less on incremental accuracy improvements alone and more on establishing SNN-based processing as a robust, edge-native computational layer for long-duration neuroengineering systems.

## 6. Wearable and Implantable Neuromorphic Platforms

Wearable and implantable neuromorphic platforms represent the system-level realization of many concepts discussed in the preceding sections. Their significance lies not primarily in the use of novel materials alone but in their ability to co-locate sensing, local memory, and lightweight computation under stringent constraints on power, latency, size, and maintenance. In neuroengineering, this integration makes them particularly relevant for real-world deployment, where continuous high-rate data streaming and reliance on remote computation are often impractical [122,123].

From this perspective, wearable and implantable neuromorphic systems are best conceptualized as edge-native architectures that bring temporal processing closer to the body, thereby enabling more autonomous, low-burden, and long-duration operation.

### 6.1. Conformable Sensing and Neuromorphic e-Skin/Textiles

**Figure 6:**
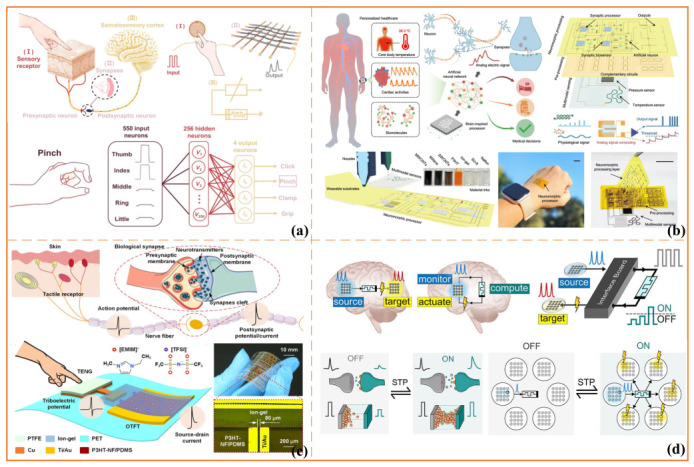
Representative examples of wearable and implantable neuromorphic platforms. (**a**) Fiber-memristor-based intelligent textile system for wearable neuromorphic sensing and hand gesture recognition, adapted from [126] under a Creative Commons Attribution 4.0 (CC BY 4.0) license; (**b**) all-printed chip-less wearable neuromorphic system for multimodal physicochemical health monitoring, adapted from [129] under a CC BY 4.0 license; (**c**) flexible tribotronic artificial synapse with bioinspired neurosensory behavior, adapted from [130] under a CC BY 4.0 license; (**d**) memristor-based monitor–compute–actuate neuromodulation interface for adaptive control of neuronal populations, adapted from [131] under a CC BY 4.0 license. Visual annotations, including arrows, circles, colors, and related graphical elements, are retained from the corresponding source panels and indicate sensing pathways, signal flows, device structures, or schematic relationships in the respective studies.

A major direction in this area is the development of conformable sensing surfaces, including neuromorphic electronic skin (e-skin) and textile-integrated nodes, that can capture pressure, shear, temperature, and deformation signals while supporting local event extraction and preliminary processing [124,125]. Compared with conventional dense sensor arrays, these platforms are particularly attractive because they are better suited for prolonged use on skin, garments, or other high-contact interfaces, where comfort, flexibility, and low user burden are essential. Representative studies have shown that soft e-skin systems can be monolithically integrated and operated at low voltage while supporting multimodal perception, neuromorphic pulse-train signal generation, and closed-loop actuation. These findings highlight the feasibility of combining tactile sensing and neuromorphic signal processing within a single conformable platform [125]. As illustrated in Figure 6a, related work on intelligent textile systems further demonstrates that fiber-based memristive devices can be woven into neuromorphic textile architectures and function as local processing elements for hand gesture recognition, suggesting a practical pathway toward wearable platforms that integrate flexibility, distributed sensing, and embedded computation [126].

From a neuromorphic perspective, the value of such front-end systems lies not in maximizing sensing density but in their ability to extract salient events from continuous interaction mechanics. Contact transitions, slip onset, abnormal load peaks, and sustained pressure hotspots can be interpreted as control-relevant events rather than as raw, high-rate data streams. This perspective is particularly useful in wearable neuroengineering systems, where sensing surfaces may serve both as monitoring interfaces for interaction safety and movement quality and as feedback-ready substrates that can be coupled with haptic or stimulation modules.

In addition, nociceptor-like neuromorphic elements have been proposed as low-power front ends for detecting noxious or overload-related cues, including bioinspired injury-response systems and wearable tactile nociceptors based on threshold-switching memristive devices [127,128]. Collectively, these studies suggest that conformable neuromorphic platforms can support not only tactile sensing but also event-driven safety monitoring under realistic operating conditions.

### 6.2. Near-Body Loops and On-Device Memory/Computation

A recurring architectural motif in wearable neuromorphic systems is the implementation of short sensing–processing–response loops in close proximity to the body. These near-body loops reduce latency and communication overhead by enabling portions of the sensing, encoding, memory, and decision-making processes to occur locally rather than relying on centralized remote computation. As shown in Figure 6b, recent work on all-printed, chip-less wearable neuromorphic systems demonstrates that multimodal physicochemical sensing, artificial synapse-based analog processing, and threshold-based decision-making can be integrated within a compact, skin-conformal platform [129]. This example illustrates that wearable neuromorphic systems can function not only as sensing layers but also as local processing and decision-support modules for physiological and biochemical monitoring. In addition, recent implantable neuromorphic sensing studies show that near-sensor computation combined with send-on-delta transmission can substantially reduce communication burden in peripheral neural recording, highlighting how local preprocessing can support low-latency and communication-efficient near-body neuroengineering interfaces [122].

Flexible and textile-oriented designs further demonstrate how local state retention and elementary computation can be embedded within wearable substrates. Representative studies integrate mechanical sensing with compact neuromorphic circuits, enabling detected events to directly drive local response pathways under low power budgets. For example, as shown in Figure 6c, flexible tribotronic artificial synapses with bioinspired neurosensory behavior can utilize contact-electrification-induced triboelectric potential to modulate synaptic transistor responses, thereby coupling mechanical stimuli with artificial synaptic dynamics in a flexible device platform [130]. Related textile-integrated, in-memory fiber memristor systems and bioinspired iontronic synapse fibers further indicate that wearable architectures can combine sensing, local memory, and elementary computation within distributed textile platforms [132,133]. Such architectures are particularly valuable because they not only detect instantaneous events but also retain recent histories of physiological signals, strain, contraction, or contact, thereby supporting trend-aware monitoring and bounded local responses.

More broadly, the significance of near-body loops lies in their ability to enable graceful degradation: even when upstream decoding or communication becomes unreliable, local event-driven responses can maintain conservative, low-latency monitoring or safety functions. In this sense, wearable neuromorphic platforms should be understood not merely as sensor carriers but as active computational layers within distributed neuroengineering systems.

### 6.3. Implantable Neuromorphic Systems

Implantable neuromorphic devices should be regarded as a selective extension of this platform logic rather than a default requirement for neuroengineering applications. Their relevance is greatest in scenarios where long-term physiological monitoring, highly localized interfacing, or continuous low-power processing justifies the additional demands of implantation, encapsulation, and long-term biocompatibility. Recent examples, such as memristive pressure-sensing systems designed for post-craniotomy intracranial pressure monitoring and pulmonary hypertension monitoring, illustrate how front-end sensing and back-end neuromorphic processing can be co-designed to support stable in vivo operation over extended periods [134,135]. At the implantable-system level, neuromorphic sensing architectures have also been developed for wireless peripheral nerve recording with integrated near-sensor processing, demonstrating how neuromorphic design principles can be extended from wearable platforms to highly localized implantable neuroengineering systems [122].

Although such systems do not directly establish benefits for every target application, they provide valuable engineering references for issues that are equally relevant to wearable deployment, including long-term stability, packaging reliability, noise-tolerant on-device inference, and constrained maintenance requirements. Beyond monitoring, memristor-based neuromodulation devices have demonstrated real-time adaptive control of neuronal populations through a monitor–compute–actuate paradigm. As shown in Figure 6d, this class of systems uses memristive elements as artificial synapses to detect relevant activity patterns and dynamically modulate coupling between neuronal populations, illustrating how implantable neuromorphic systems may evolve from passive sensing platforms toward closed-loop therapeutic interfaces [131].

In addition, neuromorphic hardware has begun to be explored in disease-oriented closed-loop applications, such as seizure detection and intervention based on memristive nanodevices [136], while broader studies in regenerative medicine have discussed the potential of neuromorphic engineering for restoring neural activity following brain injury [137]. Implantable neuromorphic systems should therefore be viewed as highly specialized platforms for selected monitoring and interfacing tasks while also providing a pathway toward future restorative and therapeutic neuroengineering applications.

To facilitate cross-study comparison, representative work in wearable and implantable neuromorphic platforms is summarized in Table 6, with emphasis on platform scenarios, neuromorphic features, system roles, and the current stage of evidence.

### 6.4. Deployment Constraints in Real-World Neuroengineering

For wearable and implantable neuromorphic platforms, the key point to emphasize is that they are increasingly functioning as platform layers rather than isolated device demonstrations. Their significance lies in bringing sensing, local memory, lightweight computation, and short response loops physically closer to the body, thereby enabling edge-native operation under realistic constraints on power, latency, form factor, and maintenance [122,123]. In this sense, the primary contribution of the current literature is not merely the introduction of new materials or flexible devices but the emergence of near-body architectures in which sensing, temporary state retention, and bounded local processing coexist within deployable substrates.

However, the main obstacle to translation is also most evident at the platform level. In wearable systems, sustained use depends on comfort, attachment stability, washability, manufacturability, and maintenance burden; in implantable systems, it depends on packaging reliability, biocompatibility, noise tolerance, and stable long-duration in vivo operation [139,140]. Beyond hardware survivability, practical value increasingly depends on interface interoperability—specifically, whether neuromorphic modules can connect to sensing, decoding, stimulation, or robotic subsystems without excessive calibration, bespoke integration effort, or hidden workflow burdens. The main bottleneck has therefore shifted from one-off demonstrations of local neuromorphic functionality to repeated, serviceable, and system-compatible use over prolonged deployment.

The next step for wearable and implantable neuromorphic platforms is therefore not merely to improve embedded computation or material performance in isolation but to establish deployment-ready evidence. Relatively few studies have systematically examined repeated wear, maintenance cycles, variable attachment conditions, long-duration in vivo use, or realistic integration with downstream decoding, stimulation, and robotic workflows [2,141]. What this field now most urgently requires is rigorous validation of durability, interoperability, safety, comfort, and maintenance under realistic neuroengineering conditions. Only then can wearable and implantable neuromorphic platforms be evaluated as practical infrastructure for long-duration neuroengineering, rather than as promising but still isolated technology demonstrations.

## 7. Neurorehabilitation as a Key Application Scenario

Neurorehabilitation represents an important translational context for neuromorphic neuroengineering, as it integrates many of the sensing, decoding, feedback, and adaptation challenges that closed-loop systems must address in practice. Rehabilitation typically unfolds over repeated sessions and relies on multimodal interactions rather than isolated, single-task events [142]. At the same time, clinically meaningful changes are often sparse and state-dependent, including variations in motor intention, fatigue, spasticity, gait phase, engagement, or contact quality [143].

These characteristics make neurorehabilitation a particularly relevant testbed for event-driven sensing, temporally structured inference, and state-dependent feedback or stimulation. In this context, its importance lies not merely in serving as another application domain but in providing a demanding environment in which the practical value of neuromorphic sensing, computation, and actuation can be evaluated in an integrated and clinically meaningful manner.

### 7.1. Why Neurorehabilitation Is a Good Testbed

A defining feature of neurorehabilitation is that it unfolds over extended time scales while relying on short, task-relevant events. Functional recovery develops across repeated sessions, yet real-time system responses are often driven by sparse transitions, including movement initiation, contact changes, fatigue-related decline, altered muscle recruitment, and fluctuations in attention or engagement. This combination of long-horizon adaptation and event-driven interaction makes rehabilitation particularly compatible with neuromorphic design principles. Event-based sensing can reduce redundancy during prolonged monitoring, spiking-based inference can preserve temporal structure in noisy physiological signals, and adaptive stimulation or feedback can be delivered at moments when state changes are most relevant, rather than according to fixed schedules [144]. Recent work on active post-stroke rehabilitation has explicitly identified intention detection and feedback technologies as two core components of active rehabilitation systems, aligning closely with the sensing–decoding–feedback framework emphasized in neuromorphic neuroengineering [145].

Neurorehabilitation is also inherently multimodal. Effective systems often require the integration of neural, muscular, kinematic, tactile, and contextual information while maintaining coherent feedback and acceptable response timing. This requirement places particular importance on architectures capable of integrating sensing and local decision-making without imposing excessive system burden. A recent systematic review and meta-analysis of BCI-based rehabilitation in stroke and spinal cord injury reported significant motor improvements, with larger gains observed when BCIs were combined with functional electrical stimulation or robotic assistance. These findings illustrate that closed-loop rehabilitation systems already tend to favor multimodal and intervention-coupled designs [144]. Neurorehabilitation therefore serves as a valuable testbed not because neuromorphic methods are already fully established in this domain but because it exposes the coordination, adaptation, and deployment challenges that such methods are specifically intended to address.

### 7.2. Opportunities in Sensing, Feedback, and Stimulation

Within neurorehabilitation, neuromorphic approaches appear most promising along three closely related directions. The first is biosignal decoding, in which SNN-based processing may support low-power and temporally informed interpretation of EEG, EMG, sEMG, pressure, or multimodal interaction signals for applications such as intention decoding, movement-state estimation, fatigue monitoring, and anomaly detection [110,111,112,113]. The second direction concerns tactile and afferent feedback, where neuromorphic sensory biointerfaces can strengthen execution–sensation coupling by translating interaction mechanics into temporally structured feedback that is more consistent with biological afferent signaling [78,79,80,81]. The third is adaptive stimulation, in which temporally patterned and state-dependent interventions may better align stimulation with evolving physiological and behavioral states during training [61,65,66,67].

Taken together, these opportunities suggest that the primary relevance of neuromorphic technology to neurorehabilitation lies not in any single component but in enabling more coherent closed-loop architectures in which sensing, decoding, feedback, and stimulation are coupled through temporally meaningful and resource-aware processing.

Recent work also supports the feasibility of this broader direction. Rehabilitation-oriented systems that combine robotics with spinal cord neuromodulation have shown that neuroprosthetic support can help organize muscle activation during robot-assisted walking and cycling in proof-of-concept studies [146]. Related research in closed-loop neuroprosthetics has further demonstrated long-term artificial sensorimotor function through the integration of tactile sensing, adaptive peripheral interfaces, and machine learning-based correction [147]. Although these studies do not yet constitute fully mature neuromorphic rehabilitation systems, they reinforce the view that neurorehabilitation provides a critical scenario for evaluating integrated sensing–feedback–intervention loops.

### 7.3. Why Translation Remains Limited

Despite this promise, translation remains limited. The current evidence base is fragmented across device demonstrations, algorithmic feasibility studies, and proof-of-concept closed-loop systems, with relatively few studies providing longitudinal validation in rehabilitation-specific contexts. Much of the strongest evidence originates from adjacent domains, such as pathological signal classification, prosthetic sensory restoration, or general wearable sensing, rather than from mature neurorehabilitation trials. Even in neural interface–based post-stroke rehabilitation, recent individual patient data meta-analyses report significant but overall modest benefits, indicating that clinical translation is encouraging yet incomplete [148]. Meanwhile, a recent systematic review of SNNs in rehabilitative wearable robotics suggests that the field remains dominated by early-stage system development rather than standardized, deployment-ready validation [149].

A further limitation is that rehabilitation-relevant targets are inherently difficult to define and annotate consistently. States such as fatigue, spasticity, engagement, compensation, and sensorimotor quality are context-dependent and less clearly labeled than canonical benchmark tasks, such as seizure or arrhythmia detection. This complicates cross-system comparison, the construction of longitudinal datasets, and the assessment of whether improvements in decoding or stimulation performance translate into meaningful rehabilitative outcomes. In addition, disorder-specific validation remains limited, and standardized endpoints that jointly capture perceptual quality, motor relevance, system robustness, and real-world usability are still lacking. Reviews of haptic-feedback interventions after stroke similarly report heterogeneous findings and insufficient evidence for definitive conclusions, despite strong conceptual promise [150].

For these reasons, neurorehabilitation should currently be viewed less as a domain in which neuromorphic methods have already achieved clear clinical superiority and more as a strategically important translational context. Its value lies in providing a demanding yet highly relevant setting in which neuromorphic neuroengineering can be evaluated not only for technical novelty but also for its capacity to support long-duration, adaptive, and practically deployable closed-loop systems.

## 8. Challenges and Future Directions

Neuromorphic neuroengineering is compelling not merely because it offers alternative hardware or algorithms but because it introduces a distinct systems-level logic for handling temporally structured, sparse, and resource-constrained biological interactions. Across domains including stimulation, sensory feedback, biosignal decoding, and wearable or implantable deployment, the promise of neuromorphic approaches lies in moving computation closer to the body, responding selectively to informative events, and integrating sensing, memory, and adaptive processing more tightly than conventional pipelines [1,2,3].

At the same time, these features introduce challenges that are often underappreciated when studies are reported primarily in terms of proof-of-concept demonstrations or isolated benchmark accuracy. The central question is therefore not whether neuromorphic systems can perform effectively under controlled conditions but whether they can remain stable, interpretable, and clinically or operationally meaningful over the extended time scales and variable environments characteristic of real-world neuroengineering applications.

### 8.1. What Neuromorphic Approaches Change in the Loop

A consistent theme across the literature is that neuromorphic approaches fundamentally alter where and when computation occurs within the system loop. Rather than relying on continuously active, centralized processing, they favor architectures in which sensing, encoding, inference, and response are distributed closer to the point of interaction and are triggered primarily by informative state changes. This shift supports temporally structured stimulation, event-aligned sensory feedback, low-power biosignal inference, and near-body processing in wearable or implantable systems [80,81,110,111,122,134,144,146].

The primary value of neuromorphic neuroengineering therefore lies less in isolated efficiency gains than in enabling always-on, low-burden functionalities such as event detection, state monitoring, temporally precise feedback, and local adaptive response. This systems-level transformation is particularly important in applications where informative events are intermittent, latency is critical, and prolonged operation must be sustained under stringent power and maintenance constraints.

### 8.2. Event Definition Drift and Nonstationary Signals

One of the most important yet under-discussed bottlenecks in neuromorphic systems is the stability of event definitions over time. Spike-based sensing and inference rely on the assumption that events remain semantically meaningful across sessions and conditions. In practice, however, event statistics can vary substantially due to factors such as threshold drift, sensor displacement, sweat-related impedance variation, motion artifacts, and inter-subject variability. In movement-related applications, heterogeneous and compensatory movement patterns further complicate the interpretation of encoded events, rendering fixed thresholding or static encoding schemes unreliable over prolonged use [104,105,106,107,108].

This issue is particularly consequential because errors in event definition propagate directly to downstream inference, feedback, or control. False events may trigger unnecessary updates, unstable bursts can bias adaptive processes, and gradual drift may silently alter control-relevant decisions without obvious signal-level failure. For this reason, encoding strategies should be explicitly designed for nonstationary operation rather than assuming stable sensing conditions.

Future work should therefore place greater emphasis on threshold adaptation, drift-aware encoding, artifact tolerance, and deployment-oriented evaluation metrics, including cross-session stability of event statistics, false-event burden, and downstream impacts on decision quality [8,114,115].

### 8.3. Device Nonidealities and Long-Term Stability

A second challenge arises from device-level nonidealities and their interaction with long-term system behavior. Memristive and synapse-like devices are attractive because they support local memory, compact analog state representation, and in-/near-memory processing; however, their responses may vary with temperature, aging, usage history, and fabrication variability [3,126]. When such devices are directly involved in sensing, inference, or adaptive control, device drift becomes more than a materials-level concern; it evolves into a systems-level issue that can influence decoding reliability, stimulation timing, and safety-critical decisions.

In wearable systems, long-term stability is further influenced by practical factors such as attachment reliability, repeated deformation, washability, skin compatibility, and maintenance burden [124,125,127,128,130,133]. Implantable systems introduce additional constraints related to encapsulation, biocompatibility, and stable in vivo performance over extended periods [122,134,135]. These considerations indicate that the translational value of neuromorphic hardware cannot be assessed solely on the basis of short-term device metrics.

Instead, future studies should systematically quantify how material- or circuit-level variability maps onto functionally meaningful system behavior, and whether calibration or compensation strategies remain compatible with low-burden deployment. A central priority will be to evaluate hardware reliability in terms of its impact on closed-loop decision-making, rather than solely in terms of isolated device performance.

### 8.4. Safe Adaptation and Bounded Personalization

Personalization is often presented as a key advantage of neuromorphic systems. Plasticity-inspired learning rules, event-driven parameter updates, and local state retention all suggest potential pathways for adaptation across sessions and users. However, in practical neuroengineering systems—particularly those involving feedback or intervention—adaptation is beneficial only if it remains bounded, interpretable, and compatible with safety constraints. Unconstrained on-device learning may amplify transient errors, lock onto unstable event statistics, or introduce behavior that is difficult to audit or predict [109,120,121,131].

The challenge, therefore, is not merely to enable adaptation but to ensure that it occurs safely. This requires bounded on-device learning, a clear separation between fast reactive adjustments and slower personalization processes, transparent failure modes, and mechanisms that enable clinician or operator oversight in safety-sensitive applications. In practice, personalization may need to proceed conservatively, with adaptation occurring only when evidence is sufficiently robust and uncertainty is acceptably low.

Reversible updates, confidence-aware adaptation, and auditable control logic are likely to be more critical for translational success than unconstrained learning performance. In this sense, the future of adaptive neuromorphic systems depends as much on governance and controllability as on plasticity itself.

### 8.5. Standardized Benchmarks and Translational Pathways

Another major limitation of the field is the lack of standardized benchmarks and well-defined translational evaluation pathways. Current evidence is often fragmented across signal types, hardware platforms, application targets, and reporting conventions, which complicates meaningful comparison. In many cases, studies emphasize proof-of-concept performance or accuracy on narrowly defined tasks while providing limited information on latency, robustness, cross-session variability, failure behavior, or operational burden [114,115]. This limitation is particularly consequential in neuroengineering, where the practical value of a system often depends more on its stability and usability under real-world conditions than on peak benchmark accuracy.

Direct cross-study comparison is further hindered by the fact that conventional and neuromorphic systems frequently report different endpoints, even when targeting similar neuroengineering functions. Conventional stimulation and rehabilitation studies typically prioritize task-level or clinical outcomes, such as movement-sequence completion, follow-up recovery metrics, or postsurgical functional status [54,65]. In contrast, neuromorphic studies more commonly report engineering-oriented metrics, including compression ratio, event-triggered latency, communication load, processor power consumption, and reconstruction fidelity [70,109,122].

Table 7 summarizes representative quantitative comparisons between conventional and neuromorphic approaches across several neuroengineering-related scenarios. The table is not intended to serve as a fully normalized benchmark across tasks; rather, it provides a concise cross-domain overview of where quantitative advantages of neuromorphic approaches have been reported and where benchmark design remains too fragmented to support firm translational conclusions.

Taken together, the most evident quantitative advantages at present lie in edge sensing, telemetry reduction, and low-burden decoding, where sparse-event representations directly reduce data movement and continuous computation. Accordingly, the strongest head-to-head evidence remains concentrated in sensing and inference pipelines rather than in fully integrated therapeutic systems. By contrast, stimulation and rehabilitation studies have demonstrated encouraging functional gains; however, their endpoints remain heterogeneous and are more frequently reported in terms of recovery or task performance than in terms of unified engineering metrics. This asymmetry is itself informative: neuromorphic neuroengineering appears to be advancing most rapidly in domains where event-driven processing maps directly onto measurable system burden, whereas translational validation still requires a more consistent linkage between engineering efficiency and clinically or behaviorally meaningful outcomes.

Application-specific metrics are therefore essential. For SNN-based biosignal processing and state decoding, these should include latency, energy consumption, drift tolerance, false-event rate, and robustness to domain shift [110,111]. For sensory interfaces and stimulation systems, reporting should extend beyond waveform or perceptual parameters to include timing reliability, state-transition fidelity, controllability, and long-term tolerance [61,70,144,146]. For wearable and implantable platforms, evaluation must additionally address usability, maintenance, comfort, washability, manufacturability, and interface interoperability [126,133]. More broadly, translational pathways will require datasets, task definitions, and reporting standards that reflect real-world neuroengineering workflows rather than solely canonical laboratory benchmarks. Without such standards, the field risks accumulating promising yet difficult-to-compare demonstrations, rather than establishing a coherent and robust evidence base for practical deployment.

### 8.6. Toward Edge-Native Closed-Loop Neuroengineering

Looking ahead, the most productive direction may be to conceptualize neuromorphic neuroengineering as an edge-native, closed-loop systems framework rather than as a collection of isolated devices or algorithms. Within such a framework, conformable or physiological sensors generate sparse event streams through robust sensing-to-spike front ends; local inference modules then interpret these events in real time; and adaptive feedback or stimulation modules respond in a manner that remains temporally aligned, bounded, and task-relevant. This closed loop is embedded within deployable, human-compatible platforms that support long-duration operation under realistic constraints on power consumption, maintenance, and interoperability [1,2,3,4,126].

Progress toward this vision will likely depend on a limited set of strategic priorities. First, event encoding must achieve robustness over extended operational time scales, rather than only under short, controlled conditions. Second, hardware reliability should be evaluated in terms of its impact on system-level decision-making, rather than solely on device-level characteristics. Third, personalization must remain bounded, reversible, and compatible with appropriate oversight. Fourth, future validation efforts should focus on integrated, edge-native systems that jointly assess sensing, inference, response, and deployment burden, rather than treating these components in isolation.

If these priorities are addressed, neuromorphic approaches may provide a credible pathway toward neuroengineering systems that are not only low-power and temporally efficient but also practically deployable, adaptive, and human-compatible over extended periods of use.

## 9. Conclusions

Neuromorphic technologies are emerging as a promising framework for neuroengineering, as they treat time, sparsity, and local adaptation as fundamental design principles. Their value lies not only in reducing energy consumption but also in enabling tighter coupling among sensing, computation, feedback, and response in ways that are more compatible with biological signaling and edge-native operation. This review has examined neuromorphic neuroengineering from four closely related perspectives: neurostimulation and adaptive actuation; tactile and sensory biointerfaces; spiking neural network (SNN)-based biosignal processing and state decoding; and wearable or implantable neuromorphic platforms. Taken together, these directions suggest that neuromorphic systems are most effective when they support low-burden, event-driven, and closed-loop functions proximal to the body, rather than merely serving as hardware substitutes for conventional computation. Neurorehabilitation represents a particularly important translational context, as it combines long-term use, multimodal sensing, adaptive intervention, and substantial deployment constraints. However, the current evidence base remains fragmented, and much of the field is still at the stage of device demonstrations and proof-of-concept systems, rather than robust translational validation. Future progress will depend on shifting from isolated component development toward integrated, edge-native systems that robustly connect sensing, local inference, adaptive feedback or stimulation, and human-compatible deployment. If these challenges are addressed, neuromorphic approaches may offer a credible pathway toward neuroengineering systems that are not only computationally efficient but also adaptive, deployable, and responsive in real-world settings.

## Figures and Tables

**Table 1:** Concise comparison of representative memristive and synapse-like device families relevant to neuromorphic neuroengineering.

Device Family	Representative Devices	Mechanism	Strengths	Limitations	Relevance
Oxide-based memristors	Metal-oxide resistive switching devices	Filamentary or interfacial resistive switching	Dense integration; crossbar compatibility	Variability; drift; nonlinear updates; endurance trade-offs	Compact synaptic arrays; in-memory weighting; edge inference [30,34]
Nitride-based artificial synaptic devices	GaN- and related nitride-based devices	Trap-mediated or interface-modulated conductance changes	Robustness; stability potential; low-power behavior	Limited diversity; analog tunability and large-scale validation still developing	Stable neuromorphic elements for harsh or long-duration operation [31,34]
Phase-change/PCM-related synaptic devices	PCM-based synaptic devices	Reversible phase transition with multilevel resistance states	Multilevel storage; strong IMC relevance	Programming overhead; thermal effects; repeated-update variability	Dense synaptic storage and analog weight programming [32,34]
Electrochemical/ iontronic/transistor-like synaptic devices	OECTs, ion-mediated oxide transistors, and electrolyte-gated synaptic transistors	Ion migration, electrochemical doping, or channel-conductance modulation	Low-voltage operation; rich synaptic dynamics; biointerface relevance	Retention, speed, uniformity, and integration remain challenging	Near-body biointerfaces; adaptive sensing; sensing-memory coupling [33,35]

**Table 2:** Representative neuromorphic hardware platforms relevant to edge and near-body neuroengineering. Reported power/energy metrics are taken from the cited sources and are not strictly normalized across tasks.

Platform	Integration	Representative Scale	Reported Power/Energy Metric	Key Hardware Feature	Neuroengineering Relevance
Loihi	14 nm CMOS	128 neuromorphic cores; 3 x86 cores; 33 MB SRAM	Over three orders-of-magnitude better energy-delay product in a benchmarked LASSO workload; energy per synaptic spike operation reported as 23.6 pJ (minimum)	On-chip learning; flexible event-driven computation	Suitable for adaptive SNN inference and low-latency closed-loop processing [38,41]
Loihi 2	Programmable digital neuromorphic processor	Microcode-programmable neural engine; up to 8192 neurons per core depending on neuron precision	Supports substantial reductions in output bandwidth and operation count in representative streaming signal-processing tasks	Richer neuron dynamics; graded spikes; broader programmability	Relevant to next-generation embedded neuromorphic signal processing and adaptive edge systems [39,42]
TrueNorth	28 nm CMOS	4096 cores; 1 million neurons; 256 million synapses	65 mW while running a typical computer vision application	Ultra-low-power large-scale neurosynaptic integration	Attractive for ultra-low-power neuromorphic sensing and classification workloads [40,41]
SpiNNaker	Digital GALS manycore SoC	18 ARM968 cores per chip; scalable to million-core systems	1 W peak chip-level power at nominal 180 MHz	Massive parallelism; real-time SNN simulation; packet-switched communication	Relevant to large-scale neuroengineering simulations and closed-loop spiking-network studies [41,43]

**Table 3:** Representative studies on neuromorphic neurostimulation and adaptive actuation.

Study	Target/Scenario	Neuromorphic or Biomimetic Feature	Loop Level	Main Point	Limitation/Evidence Stage
Xi et al. [61]	Rat hindlimb modulation during stepping	Biomimetic stimulation waveform and temporal pattern	Open-loop	Linked sciatic nerve branch stimulation to hindlimb joint modulation.	Animal feasibility study; not a closed-loop adaptive system.
Yang et al. [62]	Implantable responsive neuromodulation for pathological neural activity	Event-driven organic sensing with organic electrochemical neuron (OECN) pulse triggering	Sensor-triggered	Enabled rapid detection-triggered stimulation with low-energy operation.	Early-stage implantable study; long-term in vivo validation is still needed.
Balamur et al. [63]	Neuronal photostimulation in aqueous biointerfaces	Integrated sensing–memory–stimulation optoelectronic synapse	Interface-level	Combined photodetection, synaptic memory, and neural stimulation in a single device.	Device/interface proof of concept rather than system-level adaptive control.
Hwang et al. [65]	Closed-loop FES control with a bidirectional nerve cuff interface	Selective recording and stimulation within a bidirectional peripheral interface	Closed-loop control	Demonstrated FES control using a selectively recording and bidirectional nerve cuff interface.	Interface-level and control feasibility study; broader translational validation remains limited.
Sahai et al. [68]	Adaptive isochronous neurostimulation from a single source	Bioelectric routing for multipolar neurostimulation	Platform-level	Enabled multipolar bioelectric stimulation from a single source through an adaptive bioelectric router.	Platform-level stimulation study; sensing-coupled adaptive validation remains limited.
Lu et al. [71]	Sensory–motor coupling stimulation in SCI rats	Self-powered stimulation using a biomimetic triboelectric nanogenerator	Coupled platform	Linked joint-motion energy harvesting to sensory–motor stimulation.	Preclinical platform study; translational feasibility remains unclear.
Woods et al. [69]	Electrically stimulated neural repair scaffold	Biomimetic conductive micro-mesh interface	Non-closed-loop	Provided a conductive scaffold interface supporting stimulation-related neural responses.	Primarily an interface-support strategy rather than an adaptive stimulation system.

**Table 4:** Representative studies on neuromorphic tactile and sensory biointerfaces.

Study	Target/Scenario	Neuromorphic or Biomimetic Feature	Interface Role	Main Point	Limitation/Evidence Stage
Chen et al. [80]	Closed-loop tactile feedback for intelligent robots	Artificial organic afferent nerve integrating mechanoreceptors and a synaptic transistor	Front-end	Enabled closed-loop slip recognition and prevention in robotic manipulation.	Robotic system demonstration rather than a human neurointerface study.
Wei et al. [81]	Temporal-tactile neuromorphic sensing	Mechano-gated iontronic piezomemristor with positive/negative spike outputs	Front-end	Converted complex pressure inputs into excitatory/inhibitory neuromorphic spike trains.	Device-level demonstration focused on tactile plasticity rather than bidirectional biointerface validation.
Katic et al. [84]	Foot-sole afferent modeling for biomimetic feedback design	In silico spiking model of four major foot-sole mechanoreceptive afferent classes	Encoding pathway	Provided a computational basis for biomimetic stimulation design.	Modeling framework rather than a deployed closed-loop interface.
Valle et al. [82]	Naturalistic touch sensations via peripheral nerve stimulation	Biomimetic stimulation derived from physiologically plausible afferent codes	Encoding-to-stimulation	Produced neural responses that were closer to natural touch than regular-pulse stimulation.	Translational neuroprosthetic feedback study; broader rehabilitation validation remains limited.
Lee et al. [89]	Proprioceptive feedback in stretchable neuromorphic implants	Low-power stretchable neuromorphic nerve with integrated proprioceptive feedback	Bidirectional interface	Supported smoother and more coordinated motor output with proprioceptive feedback.	Preclinical implant study rather than a mature human sensory interface.
Cimolato et al. [91]	Proprioceptive neurostimulation encoding	Symbiotic electroneural and musculoskeletal modeling framework (ProprioStim)	Bidirectional interface	Provided an anatomically grounded framework for biomimetic proprioceptive stimulation.	Mainly a model-based encoding framework; practical long-term implementation remains open.
Sankar et al. [92]	Biomimetic prosthetic hand with tactile feedback	Three-layer neuromorphic tactile sensing in a hybrid prosthetic hand	Application system	Enabled precise and compliant grasping with naturalistic tactile sensing.	Prosthetic-system evidence is promising, but broader rehabilitation relevance remains indirect.

**Table 5:** Representative studies on SNN-based biosignal processing and state decoding.

Study	Signal/Scenario	Neuromorphic Feature	System Role	Main Point	Limitation/Evidence Stage
Barleanu et al. [104]	Force monitoring for robotic interaction	FSR-based neuromorphic sensor integrating a spiking neuron	Front-end	Converted applied force into spike-frequency outputs for robotic force sensing.	Robotic sensing demonstration rather than a biosignal decoding study.
Wang et al. [105]	Sitting posture recognition from pressure distribution	Pressure-distribution encoding for SNN-based posture classification	Front-end/inference	Demonstrated posture recognition from pressure-sensor-grid data using an SNN.	Posture-classification study; broader neuroengineering deployment relevance remains indirect.
Jiang et al. [107]	Eye tracking with event-based vision	Event camera combined with SNN processing	Event-driven inference	Enabled efficient eye tracking from sparse event streams.	Vision-based demonstration rather than a direct neural-interface application.
Sharifshazileh et al. [110]	Real-time HFO detection in intracranial EEG	Electronic neuromorphic system with online SNN-based detection	Temporal inference	Demonstrated real-time detection of high-frequency oscillations from intracranial EEG.	Application-relevant intracranial EEG study, but focused on a specific pathological event-detection task.
Yuan et al. [111]	Physiological signal processing for human–machine interfaces	VO_2_ memristor-based spike encoding and neuromorphic decision module	Hardware-oriented temporal inference	Integrated signal encoding, temporal feature extraction, and classification in one neuromorphic architecture.	Hardware-oriented proof-of-concept study; broader task generalization remains to be established.
Ma et al. [116]	Fine hand/wrist motion intention recognition from decomposed sEMG	Motor-unit spike trains processed by a residual SNN	Edge decoding	Supported fine motion intention recognition in both healthy individuals and stroke survivors.	Focused on classification performance; long-term cross-session deployment remains unclear.
Mohan et al. [109]	Wireless implantable brain–machine interfaces	Neuromorphic neural compression with AER-inspired readout	Edge pipeline	Reduced front-end data transmission while preserving spike information for downstream detection.	Front-end compression study rather than a complete closed-loop BMI validation.

**Table 6:** Representative studies on wearable and implantable neuromorphic platforms.

Study	Platform/Scenario	Neuromorphic or Biomimetic Feature	Platform Role	Main Point	Limitation/Evidence Stage
Wang et al. [125]	Soft e-skin for sensorimotor interfacing	Low-voltage soft e-skin with neuromorphic pulse-train generation	Conformable front-end	Demonstrated a soft e-skin with multimodal perception, neuromorphic signal generation, and closed-loop actuation.	Integrated soft-platform study; long-term wearable deployment remains limited.
Jiang et al. [126]	Textile neuromorphic platform for gesture recognition	Fiber memristors woven into a textile architecture	Wearable computing substrate	Showed that woven memristive textiles can support gesture recognition in intelligent textile systems.	Wearable feasibility study; long-term washability and maintenance remain unclear.
Chen et al. [133]	Neuromorphic sensorimotor textiles	Bioinspired iontronic synapse fibers for ultralow-power multiplexing	Near-body loop	Combined sensing, local memory, and elementary computation in a distributed textile platform.	Textile-level proof-of-concept study; broader neuroengineering deployment remains to be established.
Shim et al. [138]	Distributed neuromorphic cognitive skin	Stretchable synaptic-transistor-based neuromorphic skin	Wearable computational skin	Enabled distributed sensing and local neuromorphic/cognitive processing in a stretchable skin-like platform.	Wearable concept demonstration; neuroengineering-specific long-term validation remains limited.
He et al. [122]	Wireless peripheral-nerve recording	Near-sensor computation with send-on-delta transmission	Implantable sensing interface	Reduced communication burden in peripheral neural recording through near-sensor computation and send-on-delta transmission.	Focused on localized neural sensing rather than a complete closed-loop implantable system.
Wu et al. [134]	Post-craniotomy intracranial pressure monitoring	Implantable neuromorphic memristor-based pressure-sensing system	Implantable monitoring platform	Co-designed pressure sensing and neuromorphic processing for in vivo monitoring.	Targeted monitoring application; broader implantable neuroengineering generalization remains limited.
Dias et al. [131]	Adaptive neuromodulation of neuronal populations	Memristor-based monitor–compute–actuate paradigm	Closed-loop neuromodulation concept	Demonstrated real-time adaptive control of neuronal populations using memristive elements.	In vitro proof-of-concept study; not yet a deployment-ready therapeutic system.

**Table 7:** Representative quantitative comparisons between conventional and neuromorphic approaches in neuroengineering-related tasks. Values are reported as in the cited studies and are not fully normalized across datasets, cohorts, hardware platforms, or evaluation protocols.

Domain/Task	Conventional	Neuromorphic	Quantitative Comparison	Interpretation/Caveat
Peripheral-nerve sensing	Nyquist-sampling implantable sensing architectures with high transmission and storage burden [122]	Near-sensor event-based sensing with send-on-delta transmission [122]	>125× compression, 4% NRMSE, 13 μW SNN feature extraction, 28.2 μW total power in feature-extraction mode, 50 μW in full-diagnosis mode, and 10 μs temporal precision	Shows reduced data and power burden relative to conventional Nyquist-style implant sensing architectures discussed in the study, while preserving timing fidelity; this remains a system-level contrast rather than a universal implant benchmark.
Wireless neural telemetry	Full-sample or prior spike-only telemetry for large-channel implantable recording [109]	AER-inspired neural compression pipeline [109]	200–50 K compression ratio per channel, about 50× above prior work, CC ≈ 0.9, spike-detection accuracy >90%	Strong evidence that selective event transmission lowers telemetry burden with limited spike-information loss, although the contrast is centered on front-end compression rather than full implant systems.
Real-time iEEG HFO detection	Previously published template-matching Morphology Detector algorithm for HFO analysis [110]	Hardware SNN detector for HFO analysis [110]	Hardware SNN correctly predicted postsurgical outcome in 7/9 patients; overall accuracy was comparable to Morphology Detector; both reached 100% specificity	One of the clearest application-level head-to-head contrasts; neuromorphic inference reproduced established detector behavior on edge hardware, but only for a specific pathological-event task.
Post-stroke sEMG decoding	Deep residual network (ResNet) baseline for hand and wrist motion recognition [116]	Residual spiking neural network (Res-SNN) with sEMG decomposition [116]	Res-SNN >0.95 accuracy in both cohorts versus ResNet 0.84 ± 0.08/0.90 ± 0.04; in stroke survivors, Res-SNN 0.95 ± 0.03 versus CSNN 0.71 ± 0.16	Supports spike-based temporal decoding for rehabilitation-oriented intention recognition, but no long-term cross-session validation was reported.
Biofeedback nerve stimulation	Square-wave invasive electrical stimulation (Sw-iES); broader perioperative ES studies still report heterogeneous clinical endpoints [54,70]	Biomimetic biofeedback electrostimulation (Bio-iES) synchronized to physiological respiratory behavior [70]	Sw-iES versus Bio-iES: SFI −51 versus −43, CMAP 2.5 versus 3.7 mV, and NCV 24 versus 36 m/s; autograft reference −40, 4.1 mV, and 39 m/s	Suggests that physiologically matched adaptive stimulation can outperform static pulses in this task, although the reported endpoints remain task-specific and are not directly comparable to sensing- or inference-oriented engineering metrics.

## Data Availability

No new data were created or analyzed in this study. Data sharing is not applicable to this article.
